# Two new cave-dwelling species of *Triplophysa* (Cypriniformes, Nemacheilidae) from Guizhou, China

**DOI:** 10.3897/zookeys.1281.186268

**Published:** 2026-06-03

**Authors:** Ting-Ting Zhu, Feng-Hua Yuan, Ren-Yi Zhang, Ya-Hui Zhao

**Affiliations:** 1 School of Life Sciences, Guizhou Normal University, Guiyang 550025, China School of Life Sciences, Guizhou Normal University Guiyang China; 2 State Key Laboratory of Animal Biodiversity Conservation and Integrated Pest Management, Institute of Zoology, Chinese Academy of Sciences, Beijing 100101, China State Key Laboratory of Animal Biodiversity Conservation and Integrated Pest Management, Institute of Zoology, Chinese Academy of Sciences Beijing China

**Keywords:** Biospeology, cavefish, karst, morphology, phylogenetic analysis

## Abstract

Two new cave-dwelling species, *Triplophysa zhijinensis***sp. nov**. and *Triplophysa dafangensis***sp. nov**., are described from karst caves in Guizhou Province, southwestern China. *Triplophysa zhijinensis***sp. nov**. is distinguished from other hypogean congeners by body scaleless, dorsal with pigment spots, lateral line complete; eye degenerated into small black spot or disappears, anterior nostril with barbel-like tip; distal margin of dorsal fin truncate; tip of pelvic fin not reaching to anus. *Triplophysa dafangensis***sp. nov**. is distinguished from other hypogean congeners by body naked, without skin pigmentation, lateral line complete; eye reduced, with diameter of head length 5.3–7.1%; outer rostral barbel reaching to or beyond posterior margin of posterior nostrils; distal margin of dorsal fin truncate; tip of pectoral fin not reaching to pelvic-fin origin. To further validate their taxonomic status, mitochondrial cytochrome *b* (Cyt *b*) gene sequences were used to reconstruct phylogenetic relationships and assess genetic distances. The molecular data corroborate the morphological findings and support the recognition of both species as distinct evolutionary lineages. These discoveries highlight the underestimated diversity of *Triplophysa* in subterranean habitats and emphasize the ecological importance of karst groundwater systems as sheltered habitats for specialized cave-adapted fauna.

## Introduction

China is the most biodiverse country in the world regarding cavefish species, with more than 190 valid species, more than half of which are stygobitic species (Maldonado et al. 2020). These fish are primarily found in the karst regions of southwestern China, particularly in the provinces of Guizhou, Guangxi, and Yunnan (Zhao et al. 2022; Cao et al. 2025; Shi et al. 2025). Among these regions, Guizhou is especially notable as a hotspot for cavefish diversity, featuring a significant number of stygobitic species. The province’s unique karst topography, with its extensive network of limestone caves and underground rivers, provides an ideal environment for the evolution and specialization of cavefish species (Li et al. 2022; Luo et al. 2023). Guizhou’s rich aquatic ecosystems and distinctive hydrological conditions have made it a critical area for understanding the distribution and ecological significance of cavefishes in China.

*Triplophysa* is one of the most species-rich and morphologically diverse genera of Nemacheilidae and represents a major component of the ichthyofauna of the Qinghai–Tibet Plateau (Zhang and Zhao 2016). At present, more than 170 valid species are recognized worldwide (Fricke et al. 2026). Species of *Triplophysa* are generally characterized by an elongated body, three pairs of barbels, a well-developed lower jaw, and various degrees of scale reduction (Zhu 1989). In addition to inhabiting high-altitude surface waters, some species of the genus also occur in the karst regions of southern China. The well-developed cave and subterranean river systems in these areas provide stable underground habitats for some *Triplophysa* species, thereby facilitating the evolution of cave-dwelling lineages. The genus *Triplophysa* is one of the most diverse groups of cave-dwelling fishes in China, particularly in karst regions such as Guizhou (Zhu et al. 2025). This genus includes ~ 46 cave-dwelling species, many of which are regionally endemic (Cao et al. 2025; Shi et al. 2025; Zhu et al. 2025; Dong et al. 2026; Yuan et al. 2026). Members of *Triplophysa* exhibit a wide spectrum of cave-related adaptations, making them a key model group for understanding evolutionary divergence and morphological plasticity in subterranean environments. Despite their ecological significance, many cave-dwelling species of *Triplophysa* remain poorly described or undiscovered, highlighting the need for continued taxonomic and biodiversity surveys in the region.

During recent ichthyological investigations in karst caves of northwestern Guizhou, China, we collected specimens representing two previously unknown cave-dwelling species of *Triplophysa*. In this study, we describe these two new species based on detailed morphological characteristics and molecular evidence.

## Materials and methods

### Sample collection

All samples were collected using hand nets. After collection, the fish were euthanized in compliance with the relevant laws of the Chinese Laboratory of Animal Welfare and Ethics (GB/T 35892-2018). Tissue samples for molecular analyses were taken immediately after collection and preserved in anhydrous ethanol at −20 °C, while the type specimens were fixed in 10% formalin for one week and subsequently transferred to 75% ethanol for permanent preservation. The specimens were deposited at the School of Life Sciences, Guizhou Normal University (**GZNUSLS**), Guiyang City, Guizhou Province, China. A total of 153 specimens of 25 cave-dwelling species of the genus *Triplophysa* were used for the comparison of morphological characteristics of the new species. Detailed information is provided in the Appendix 1.

### Morphological measurements and analyses

The method of counts and morphometric measurements, related terminology follow explanations given in Li (2018). Point-to-point measurements were taken on the left side of the fish specimens using a vernier caliper and recorded to the nearest 0.1 mm. Body and head-related measurements were expressed as percentages of standard length (SL) and head length (HL), respectively. The vertebral counts of two specimens were obtained through a micro-CT scan (Bruker Skyscan 1276).

### DNA extraction, PCR, and sequencing

Genomic DNA was isolated from muscle samples using a conventional high-salt protocol (Aljanabi and Martinez 1997) with proteinase K digestion. DNA quality verification included 1% agarose gel electrophoresis for integrity assessment and spectrophotometric quantification using a Biotek Epoch 2 system (Biotek Instruments, Inc., Winooski, VT, USA). Amplification of the mitochondrial Cyt *b* gene employed primers L14724 (5’-GACTTGAAAAACCACCGTTG-3’) and H15915 (5’-CTCCGATCTCCGGATTACAAGAC-3’) (Xiao et al. 2001) in 35 µL PCR reactions containing: 17.5 µL 2× Taq Plus MasterMix (CoWin Biosciences, Beijing, China), 14.5 µL ultrapure water, 1 µL template DNA (100 ng/µL), and 1 µL each primer (10 µM). Thermal cycling parameters included: 5 min initial denaturation at 95 °C; 35 cycles of 95 °C/1 min, 54 °C/30 s, 72 °C/1.5 min; final extension at 72 °C/10 min. Amplification success was confirmed by agarose gel electrophoresis using DL2000 markers (TaKaRa, Beijing, China). PCR products were commercially sequenced by Sangon Biotech (Shanghai, China).

### Molecular data analysis

A total of 64 Cyt *b* sequences were used for phylogenetic reconstruction, including five newly generated in this study and 59 retrieved from NCBI GenBank (https://www.ncbi.nlm.nih.gov/) (Table 1). Bayesian inference (BI) analyses were conducted using MrBayes v. 3.2.6 (Ronquist et al. 2012), implemented in PhyloSuite v. 1.2.3 (Zhang et al. 2020). Four Markov Chain Monte Carlo (MCMC) chains were run for 5 million generations, with trees sampled every 1,000 generations and the initial 25% discarded as burn-in. A majority-rule consensus tree was generated to estimate Bayesian posterior probabilities (BPP). Phylogenetic trees were visualized and annotated using the Interactive Tree of Life (iTOL) platform (https://itol.embl.de/). Pairwise genetic distances were calculated under the Kimura 2-Parameter (K2P) model using MEGA v. 7.0 (Kumar et al. 2016).

**Table 1.** Complete list of GenBank accession numbers for all sequences included in the present molecular phylogenetic analyses.

Species		Voucher	GenBank accession numbers
1	*Barbatula barbatula*	/	KP715096
2	*Claea dabryi*	KIZ 2009002750	MG238215
3	*C. minibarba*	IHB 2017097698	OP750015
4	*C. wulongensis*	T20	OQ754129
5	*Homatula berezowskii*	FS-2014-Y03	NC_040302
6	*H. pycnolepis*	No. 20080819953	NC_056344
7	*Triplophysa alticeps*	H14	OP616079
8	*T. angeli*	/	NC_065113
9	*T. anlongensis*	GZNU 20230112003	OQ754140
10	*T. anterodorsalis*	F3894	MG725417
11	*T. baotianensis*	GZNU 20180421006	OQ241181
12	*T. bleekeri*	/	NC_018774
13	*T. brevicauda*	KIZ 050422005	MG238301
14	*T. cehengensis*	GZNU 20230109003	OQ754134
15	*T. dafangensis* sp. nov.	GZNUSLS202501288	PZ301237
16	*T. dafangensis* sp. nov.	GZNUSLS202501289	PZ301238
17	*T. dorsalis*	F740	MG725413
18	*T. erythraea*	/	NC_088519
19	*T. guizhouensis*	GZNUSLS202502020	PV394924
20	*T. huapingensis*	T13	OQ754125
21	*T. langpingensis*	T10	OQ754122
22	*T. lixianensis*	/	NC_030521
23	*T. longliensis*	SWU2016090300	MW582825
24	*T. macrocephala*	T11	OQ754123
25	*T. markehenensis*	F3893	MG725416
26	*T. nandanensis*	/	MW582824
27	*T. nanpanjiangensis*	GZNUSLS202407050	PV394925
28	*T. nasobarbatula*	GZNU 20220313011	OQ241176
29	*T. nayongensis*	GZU001	PX935248
30	*T. nujiangensa*	IHB201315814	KT213598
31	*T. panzhouensis*	GZNU 20220513003	OQ754121
32	*T. pappenheimi*	/	NC_033972
33	*T. qingzhenensis*	IHB 201911150005	MT700459
34	*T. qini*	/	ON528184
35	*T. qiubeiensis*	T15	OQ754127
36	*T. robusta*	/	NC_025632
37	*T. rongduensis*	GZNU 20230110003	OQ754137
38	*T. rosa*	SWU10100503	JF268621
39	*T. rotundiventris*	F2077	MG725402
40	*T. sanduensis*	SWU20170613001	MW582822
41	*T. siluroides*	/	EF212443
42	*T. stenura*	/	OR916123
43	*T. stewarti*	/	NC_030506
44	*T. stoliczkai*	F2565	MG725410
45	*T. strauchii*	CF736	KX373854
46	*T. tenuis*	IHB0917490	KT224363
47	*T. tianeensis*	/	MW582826
48	*T. tianxingensis*	GZNUSLS202309182	PV394926
49	*T. tibetana*	NWIPB1106069	KT224364
50	*T. venusta*	/	NC_029330
51	*T. weiheensis*	CF3569	KY781403
52	*T. wenshanensis*	/	PP661512
53	*T. wudangensis*	T22	OQ754131
54	*T. wuweiensis*	IHB201307124	KT224365
55	*T. xiangxiensis*	/	JN696407
56	*T. xiuwenensis*	GZNUSLS202306089	PV394922
57	*T. xuanweiensis*	ASIZB223820	OL675198
58	*T. yaluwang*	GZNU20240118005	PQ117067
59	*T. yangi*	/	PQ356185
60	*T. zhenfengensis*	GZNUSLS202311039	PV394927
61	*T. zhijinensis* sp. nov.	GZNUSLS202410063	PZ301239
62	*T. zhijinensis* sp. nov.	GZNUSLS202410064	PZ301240
63	*T. zhijinensis* sp. nov.	GZNUSLS202410065	PZ301241
64	*T. ziyunensis*	GZNU20230529003	PQ117069

## Results

### Triplophysa zhijinensis sp. nov.

Taxon classificationAnimaliaCypriniformesNemacheilidae

1EEDCEA0-F2E4-5F88-AFC5-8514888D2511

https://zoobank.org/1D487C67-E7BD-4AFD-830E-C70D466E8398

**Figure 1.**
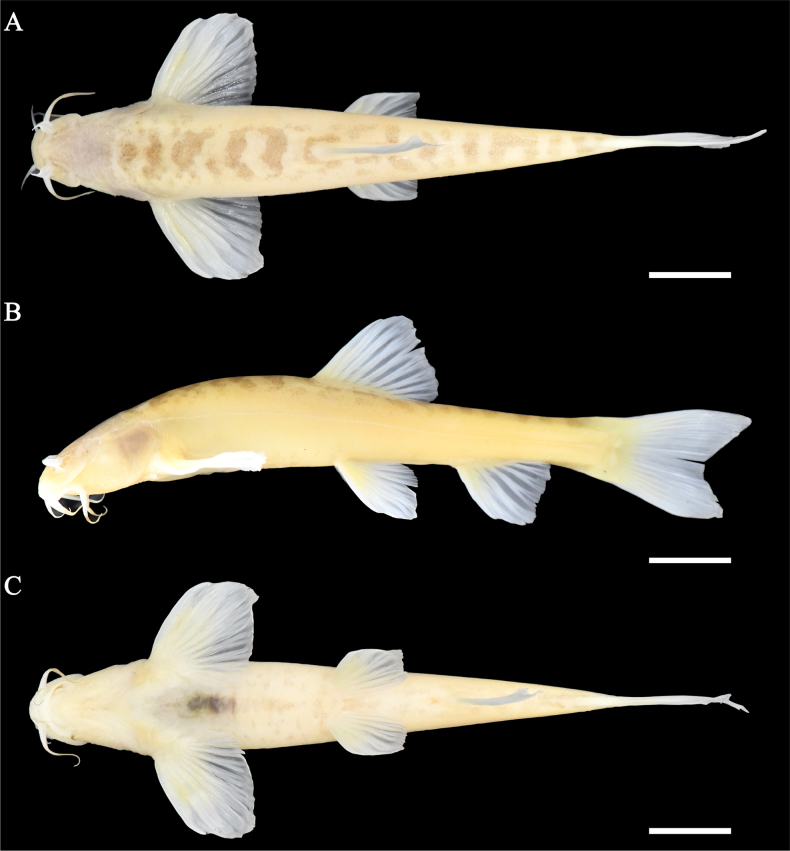
*Triplophysa zhijinensis* sp. nov., holotype, GZNUSLS202410072 74.1 mm SL. **A**. Dorsal view; **B**. Lateral view; **C**. Ventral view.

**Figure 2.**
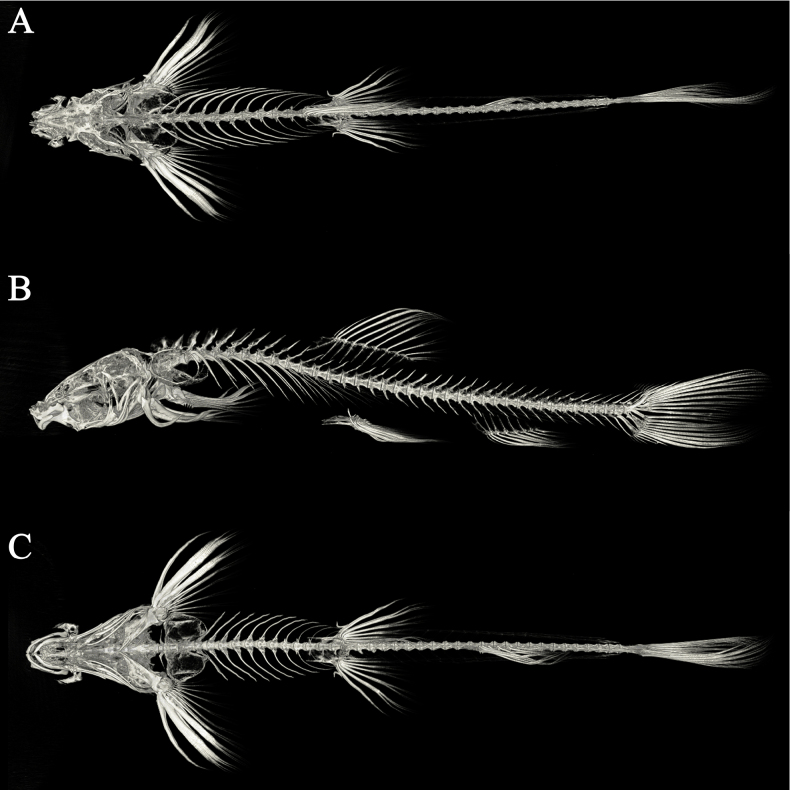
*Triplophysa zhijinensis* sp. nov., holotype, micro-CTgraph. **A**. Dorsal view; **B**. Lateral view; **C**. Ventral view.

**Figure 3.**
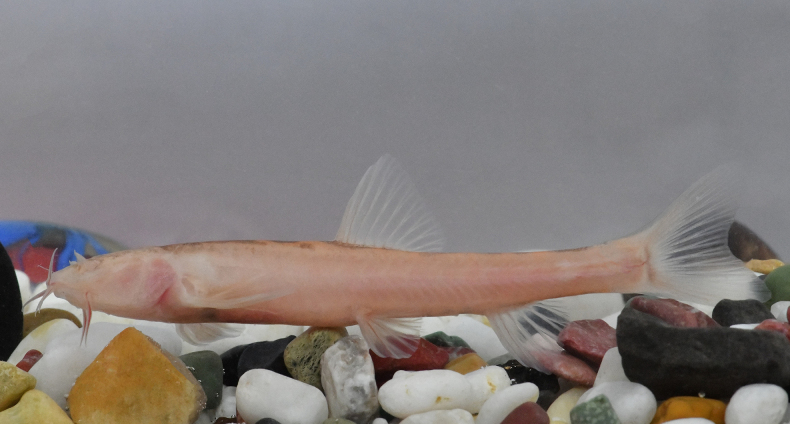
Live specimens of *Triplophysa zhijinensis* sp. nov.

**Figure 4.**
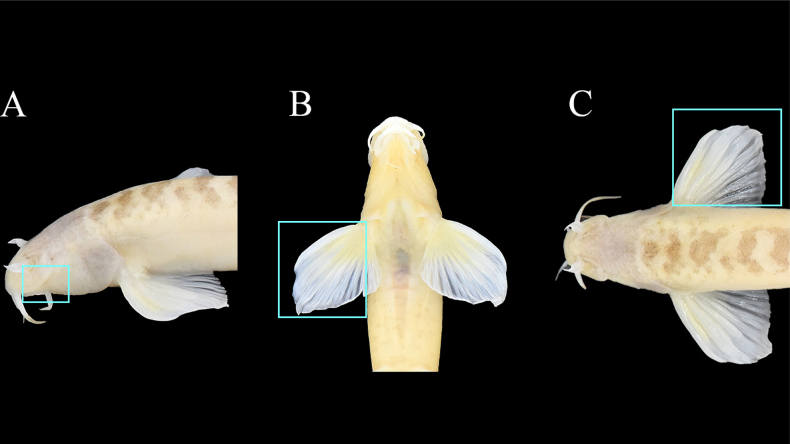
Sexual dimorphism of *Triplophysa zhijinensis* sp. nov., holotype. **A**. Lateral view show the head; **B**. Ventral view show the pectoral fins and abdominal region; **C**. Dorsal view with an inset show the thickened and broadened pectoral fin morphology. Boxes indicate regions highlighting sexual dimorphism.

**Figure 5.**
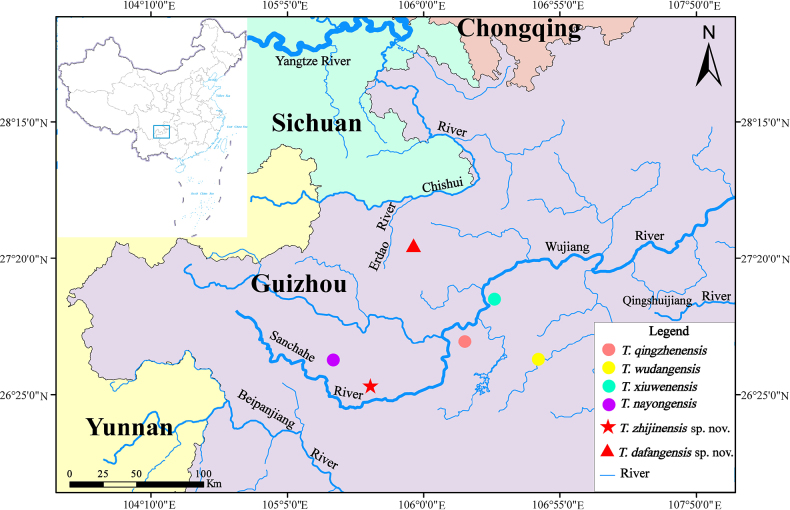
Type localities of the two new species from Guizhou Province and river systems showing the distribution of *Triplophysa* species in the same and adjacent drainages. Red triangles indicate the type locality of *Triplophysa dafangensis* sp. nov., and red stars indicate the type locality of *Triplophysa zhijinensis* sp. nov. Circles in different colors represent other *Triplophysa* species distributed in adjacent river systems. Blue lines denote rivers.

Figs 1, 2, 3, 4, 5, Tables 2, 3

#### Type material.

***Holotype***. • GZNUSLS202410072 (Fig. 1), 74.1 mm SL, collected by Ren-Yi Zhang, Ting-Ting Zhu, and local people on October 30, 2024, in Thirteen Bay Village, Xiongjiachang Town, Zhijin County, Guizhou Province, China (26.4766°N, 105.6401°E; ca 1271 m; Fig. 5). ***Paratypes***. • Four specimens from the same locality as the holotype. GZNUSLS202410063–065, 69.0–86.6 mm SL, GZNUSLS202410073, 60.7 mm SL, the collection data are the same as that of the holotype specimen.

**Table 2.** Morphometric data of *Triplophysa zhijinensis* sp. nov. and *Triplophysa dafangensis* sp. nov.

	*Triplophysa zhijinensis* sp. nov.		*Triplophysa dafangensis* sp. nov.	
	Holotype	Range (Mean, SD)	Holotype	Range (Mean, SD)
		(*n* = 5)		(*n* = 7)
Total length, mm	91.8	75.1–106.8	85.6	46.6–85.6
SL, mm	74.1	60.7–86.6	73.0	37.6–73.0
HL, mm	17.4	15.4–20.7	18.3	10.0–18.3
**In percent SL%**				
Body height	15.7	13.0–15.7 (14.5, 1.0)	12.9	12.9–15.8 (14.6, 0.9)
Body width	14	11.9–14.0 (12.6, 0.8)	10.9	9.8–12.3 (11.2, 1.0)
Predorsal length	48.8	47.6–51.1 (49.3, 1.5)	53.4	52.5–55.0 (53.8, 0.9)
Dorsal fin length	21.4	19.9–21.4 (20.8, 0.6)	19	18.3–21.4 (20.0, 1.1)
Dorsal fin base length	14.9	11.3–14.9 (12.9, 1.4)	12.9	10.8–12.9 (12.1, 0.7)
Prepelvic length	53.2	51.4–54.3 (52.6, 1.1)	54.9	53.3–55.7 (54.3, 0.8)
Pelvic fin length	17.9	16.4–18.3 (17.5, 0.9)	14.7	14.7–16.6 (15.9, 0.7)
Pelvic fin base length	4.3	3.3–4.3 (3.9, 0.4)	2.9	2.8–4.0 (3.2, 0.4)
Preanal length	74.1	72.6–76.6 (74.3, 1.6)	72.6	72.0–75.8 (73.8, 1.2)
Anal fin length	17.6	15.2–18.6 (17.0, 1.3)	16.4	16.1–17.2 (16.8, 0.4)
Anal fin base length	10.6	7.9–10.6 (9.1, 1.0)	9.9	8.8–11.2 (9.9, 0.8)
Prepectoral length	23.0	23.0–25.8 (24.4, 1.1)	24.3	23.6–28.1 (25.2, 1.6)
Pectoral fin length	21.5	20.4–21.5 (20.8, 0.4)	18.1	18.1–21.7 (20.5, 1.3)
Pectoral fin base length	5.6	5.3–6.4 (5.7, 0.4)	3.9	1.5–4.4 (3.6, 1.0)
Preanal length	69.5	69.2–72.4 (70.4, 1.4)	69.3	68.7–70.6 (69.7, 0.7)
Caudal peduncle length	16.4	16.4–19.3 (18.1, 1.5)	17.3	16.5–19.7 (17.5, 1.1)
Caudal peduncle depth	8.4	6.6–8.4 (7.7, 0.7)	6.9	6.3–7.6 (7.1, 0.4)
**In percent HL%**				
Head height	62.2	52.2–62.2 (56.7, 3.5)	53.8	50.2–62.0 (56.0, 3.6)
Head width	69.4	59.3–71.2 (65.4, 4.9)	62.9	62.9–70.0 (66.6, 2.6)
Inner rostral barbel length	29.7	21.0–29.7 (23.9, 3.5)	25.8	19.3–26.5 (22.9, 3.3)
Outer rostral barbel length	58.9	45.4–58.9 (50.2, 5.4)	42.8	38.6–52.2 (44.5, 5.0)
Maxillary barbel length	43.9	35.1–43.9 (40.6, 3.3)	32	30.8–38.2 (33.9, 2.7)
Eye diameter	1.3	1.3/2.9 (2.1, 1.1)	5.3	5.3–7.1 (6.2, 0.8)
Interorbital width	35.3	35.1/35.3 (35.2, 0.1)	33.4	33.4–38.4 (35.5, 2.0)
Distance between posterior nostrils	24.3	22.2–28.3 (24.8, 2.2)	23.7	19.4–25.9 (23.3, 2.7)
Pre-anterior nostril length	21.8	21.2–27.5 (24.5, 2.9)	27.2	22.5–28.0 (26.1, 1.9)
Postocular head length	54.8	48.2–54.8 (51.5, 4.6)	49.5	49.5–54.3 (52.3, 1.8)
Snout length	47.9	42.2–47.9 (45.1, 4.0)	45.7	36.4–47.3 (42.4, 3.7)

**Table 3.** Morphology of two new species described in this paper was compared with that of 44 known species of hypogean group of *Triplophysa*.

ID	Species	Skin pigmentation	Eye diameter (% HL)	Vertebrae	Posterior chamber of air bladder	Dorsal fin rays	Anal fin rays	Caudal fin rays	CPL/CPD	Ventral fin tip reaching to anus
**1**	*T. aluensis*	Absent	5.6	–	Degenerated	iii, 7	iii, 5	13	2.8	No
**2**	*T. anlongensis*	Whole body	5.1–9.3	4 + 37	Degenerated	iii, 8	iii, 5	16	1.7	No
**3**	*T. anshuiensis*	Dorsal	Absent	–	Developed	iv, 7–8	ii, 6	14	1.59	Yes
**4**	*T. baishuijiangensis*	Absent	2.9–6.1	4 + 34	Developed	iii, 7	ii, 5	15–16	2.3–2.7	Yes
**5**	*T. baotianensis*	Whole body	14.0–15.0	–	Degenerated	iii, 6–7	ii, 5	11–13	1.7–2.3	No
**6**	*T. cehengensis*	Absent	1.5–2.2	4 + 35	Developed	iv, 9	iii, 5	16	2	Yes
**7**	*T. erythraea*	Absent	Absent	–	Developed	ii, 8	i, 6	17	1.9–2.4	Yes
**8**	*T. fengshanensis*	Absent	Absent	–	–	ii, 8	ii, 6	16	2.4–2.6	No
**9**	*T. flavicorpus*	Whole body	5.1–6.8	4 + 34	Degenerated	iii, 10	iii, 6–7	16	1.7	Yes
**10**	*T. gejiuensis*	Absent	Absent	–	Developed	iii, 7–8	iii, 4–6	14–15	1.4	Yes
**11**	*T. guizhouensis*	Whole body	9.4–12.1	–	Developed	iii, 8	iii, 6	14	1.7	No
**12**	*T. huapingensis*	Whole body	10.4–14.3	–	Degenerated	iii, 8–9	iii, 5	16	1.7–1.8	No
**13**	*T. langpingensis*	Absent	2.7–5.9	–	–	iii, 7–8	iii, 5–6	14	1.55	Yes
**14**	*T. longipectoralis*	Whole body	11.8–16.4	4 + 35	Degenerated	iii, 8	iii, 5–6	14–15	1.4	Yes
**15**	*T. longliensis*	Whole body	9.5–11.5	4 + 38	Developed	iii, 8	iii, 5	15–16	2.43	Yes
**16**	*T. luochengensis*	Whole body	7.5–8.6	4 + 33–34	Degenerated	iii, 8	ii, 6	16–17	1.6	No
**17**	*T. macrocephala*	Whole body	3.6–8.0	–	Degenerated	iii, 7–9	iii, 5–6	15–17	1.95	Yes
**18**	*T. nandanensis*	Whole body	11.1–21.3	4 + 36	Degenerated	iv, 8	iv, 5	14–16	1.69	No
**19**	*T. nanpanjiangensis*	Whole body	12.0–16.5	4 + 38	Degenerated	iii, 7–8	ii, 5	16	1.7–2.3	No
**20**	*T. nasobarbatula*	Whole body	9.1–13.3	4 + 36	Degenerated	iii, 8	iii, 5	15	1 .4–1.8	Yes
**21**	*T. nayongensis*	Absent	Absent	4 + 35	Developed	iii, 8	iii, 5–6	12–14	2.3–2.9	Yes
**22**	*T. panzhouensis*	Whole body	7.0–11.0	4 + 35	Degenerated	iv, 7–8	iii, 5	16	2.2	No
**23**	*T. posterodorsalus*	Absent	Absent	–	–	iii, 6	ii, 4	15	3.33	No
**24**	*T. qingzhenensis*	Whole body	2.1–4.4	4 + 36	Degenerated	iii, 7–8	iii, 5	14	2.25	No
**25**	*T. qini*	Absent	Absent	4 + 34–35	–	ii, 8	ii, 5	14–16	2.36	Yes
**26**	*T. qiubeiensis*	Absent	Absent	4 + 35	Degenerated	iii, 7	iii, 5	14–15	2.0–2.8	Yes
**27**	*T. rongduensis*	Whole body	7.2–14.7	4 + 39	Degenerated	iv, 9	iii, 5	16	1.62	No
**28**	*T. rosa*	Absent	Absent	–	–	iii, 9	iii, 6	14	2.3	Yes
**29**	*T. sanduensis*	Whole body	11.9–15.4	4 + 37	Degenerated	ii, 8–9	i, 5	17–18	1.58	No
**30**	*T. shilinensis*	Absent	Absent	–	Degenerated	iii, 7	iii, 5	14	2.47	No
**31**	*T. tianeensis*	Whole body	3.0–5.9	4 + 35	Degenerated	iii, 6–7	iii, 6	15–16	1.84	No
**32**	*T. tianlinensis*	Absent	Absent	–	Degenerated	iv, 8–9	iii, 6	15–16	1.55	Yes
**33**	*T. tianxingensis*	Whole body	4.2–6.7	4 + 38	Developed	iii, 8	ii, 5	16	2	No
**34**	*T. wenshanensis*	Whole body	5.1–6.4	4 + 38~39	Normally developed	iii, 8–10	iii, 6–8	17–19	2.2–2.8	No
**35**	*T. wudangensis*	Whole body	5.1–6.5	4 + 34	Degenerated	iii, 7	iii, 5	14	2.56	No
**36**	*T. xiangshuingensis*	Whole body	7.5	–	Degenerated	iii, 6	iii, 5	14	1.9	No
**37**	*T. xiangxiensis*	Absent	Absent	–	Developed	iii, 8	iii, 6	16	2.37	Yes
**38**	*T. xichouensis*	Whole body	Absent	4 + 36	Developed	iii, 8	ii, 6	16	2.8	Yes
**39**	*T. xingyiensis*	Whole body	9.1–17.3	4 + 36	Degenerated	ii, 7	i, 5	16	1.9–2.2	No
**40**	*T. xiuwenensis*	Absent	4.6–6.7	4 + 37	Degenerated	iii, 8	iii, 6	14–15	2.1–2.5	No
**41**	*T. xuanweiensis*	Absent	Absent	–	Well developed	iii, 7–8	iii, 5	17–18	2.02	Yes
**42**	*T. yaluwang*	Whole body	4.6–6.1	4 + 36	Degenerated	iii, 7	iii, 5	14	2.5	Yes
**43**	*T. yangi*	Absent	2–5.3	4 + 34~35	Well developed	iii, 7–9	iii, 5–7	15–18	1.7–1.9	Yes
**44**	*T. yunnanensis*	Whole body	7.2–8.3	–	Degenerated	iii, 7	iii, 5	15–16	1.8–2.1	No
**45**	*T. zhenfengensis*	Whole body	7.1–16.7	4 + 36	Degenerated	iii, 7	iii, 5	14–15	1.6–2.0	No
**46**	*T. ziyunensis*	Whole body	2.4–4.9	4 + 35	Degenerated	iii, 8	iii, 5	16	2.34	Yes
**47**	*T. dafangensis* sp. nov.	Absent	5.3–7.1	4 + 39	Degenerated	iii, 7–8	iii, 6–7	14–16	2.3–2.8	Yes
**48**	*T. zhijinensis* sp. nov.	Present	Absent or 1.3–2.9	4 + 35	Degenerated	iii, 8	iii, 5–6	14–15	2.0–2.9	No

#### Diagnosis.

*Triplophysa zhijinensis* sp. nov. can be distinguished from all other species in the genus *Triplophysa* by the following combination of characteristics: body scaleless, dorsal with pigment spots in preserved specimens, lateral line complete; eyes are either absent or have degenerated into a small black spot with a diameter of 1.3–2.9% of HL; anterior nostril with barbel-like tip; distal margin of dorsal fin truncate; tip of pelvic fin not reaching to anus; dorsal fin 8, anal fin 5–6, and caudal fin 14–15 branched fin rays; vertebrae 39 (Fig. 2).

#### Description.

D iii, 8; A iii, 5–6; P i, 9; V ii, 5–6; C 14–15; vertebrae 4+35 (*n* = 1).

***Body*** extended, forequarters subcylindrical, hindquarters laterally compressed, dorsal profile of head gradually rising. Dorsal profile of body convex. Abdominal profile almost straight from end of muzzle to beginning of anal fin, slightly concave from the beginning of the anal fin. Maximum body depth reached before the origin of the dorsal fin, deepest body depth 13.0–15.7% of SL; body slightly tapering toward the caudal fin base. Body smooth and scaleless; lateral line complete. Cephalic lateral-line system with 4+5 supraorbital pores, 6 infraorbital pores, 3 supratemporal pores, and 11 preopercular pores.

***Head*** robust, width slightly greater than depth, 22.4–25.7% of SL. Snout pointed; eyes highly degenerated–absent in three specimens examined, and two individuals retaining eyes as small black spots, eye diameter 1.3 and 2.9% of HL. Interorbital width 35.1/35.3% of head length. Anterior and posterior nostrils connected; posterior nostril larger than anterior. Anterior within nasal flap, extended into barbel-like tip.

***Mouth*** inferior; mouth corner located below anterior nostril. Upper and lower lips thick; upper lip with fine papillae; lower lip centrally notched, divided into left and right lobes. Upper jaw without dentary processes; margin of lower jaw exposed, spatulate in shape, distinct V–shaped median incision on lower lip. Three pairs of barbels, all elongated: one pair each of inner rostral, outer rostral, and maxillary barbels. Inner rostral barbels 21.0–29.7% of HL, extending posteriorly to nostrils; outer rostral barbels well-developed, 45.4–58.9% of HL, extending beyond base of maxillary barbels; maxillary barbels 35.1–43.9% of HL, reaching beyond posterior margin of nostrils, exceeding half the distance to the opercular bone. Posterior chamber degenerated, with the bony capsule forming a dumbbell shape.

Dorsal fin origin located approximately at the midpoint between snout tip and caudal fin base, 19.9–21.4% of SL; fin margin truncate, with the first branched ray longest. Fin ray tips extend to the vertical through the anus. Anal fin well-developed, with concave posterior margin, 15.2–18.6% of SL; fin rays do not reach caudal fin base. Pectoral fins well-developed, 20.4–21.5% of SL, extending horizontally fin rays reach approximately halfway between pectoral and pelvic fin origins. Pelvic fins well developed, 16.4–18.3% of SL; origin slightly posterior to dorsal-fin origin, the tip of pelvic fin not reaching the anus. Anus positioned close to anal fin origin. Caudal fin forked, upper lobe longer than lower, tip slightly pointed. Adipose-like fin folds with slight keel along dorsal and ventral edges of caudal peduncle, continuous with caudal fin.

#### Coloration.

Live individuals in natural habitat exhibit depigmented body surfaces. After rearing under natural light for two days, pigmentation appears as irregular patches on dorsal surface (Fig. 3). All fins colorless. After fixation in 10% formalin, ventral body turns milky white; body and fin rays whitish, with light brown patches on head and dorsal body; remaining body areas pale yellow. All fin rays milky white, bases pale yellow (Fig. 1).

#### Sexual dimorphism.

In one male specimen (GZNUSLS202410072, 74.1 mm SL), microvillous microspinules were present on the anterior margin of the eye, outside of nostrils, and outside of rostral barbel area (Fig. 4A); and second to fourth branched pectoral-fin rays are thickened and broadened, with their dorsal surfaces covered by numerous breeding tubercles (Fig. 4B, C).

#### Distribution.

The new species was collected from an underground river in Xiongjiachang Town, Zhijin County, which is connected to the Sanchahe River, a tributary of the upper reaches of the Wujiang River, and finally joins the Yangtze River (Fig. 5).

#### Etymology.

The specific name refers to the type locality (county) of the new species: Zhijin County and the Latin suffix (ensis). We propose the Chinese name ‘Zhī jīn Gāo yuán qiū’ (织金高原鳅).

### Triplophysa dafangensis sp. nov.

Taxon classificationAnimaliaCypriniformesNemacheilidae

91A806BD-2E3C-5CC8-B1E3-3F3BDB46EB51

https://zoobank.org/A2A19271-19E6-4648-B42A-A03BA5E2D62C

**Figure 6.**
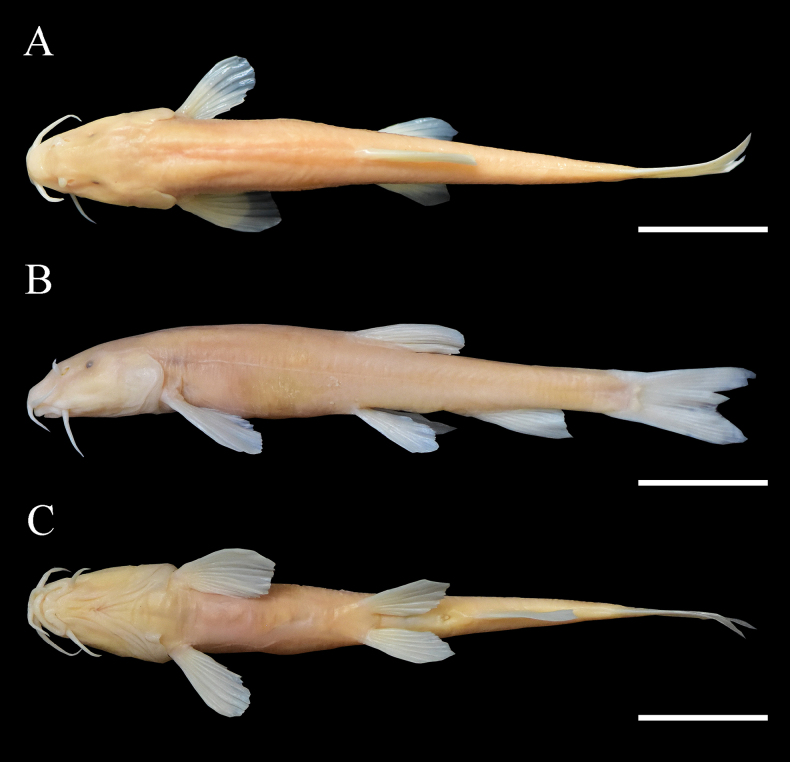
*Triplophysa dafangensis* sp. nov., holotype, GZNUSLS202501291, 73.0 mm SL. **A**. Dorsal view; **B**. Lateral view; **C**. Ventral view.

**Figure 7.**
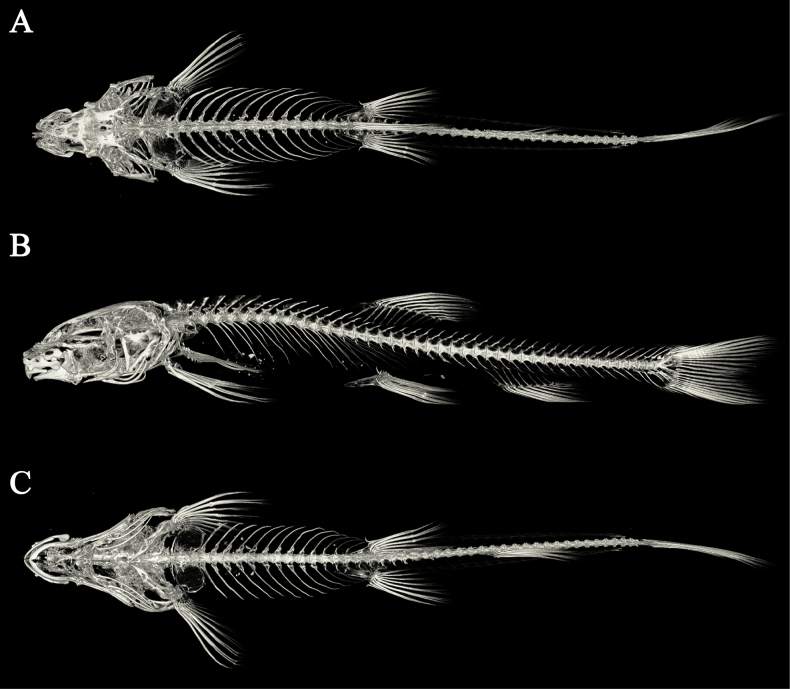
*Triplophysa dafangensis* sp. nov., holotype, micro-CTgraph. **A**. Dorsal view; **B**. Lateral view; **C**. Ventral view.

**Figure 8.**
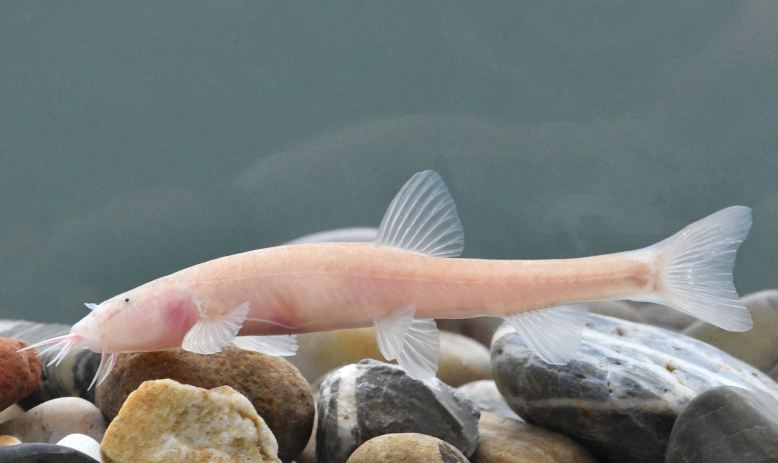
Live specimens of *Triplophysa dafangensis* sp. nov.

Figs 5, 6, 7, 8, Tables 2, 3

#### Type material.

***Holotype***. • GZNUSLS202501291 (Fig. 6), 73.0 mm SL, collected by Ren-Yi Zhang, Ting-Ting Zhu, Lei-Shan Wang, Feng-Hua Yuan, and local people on January 7, 2025, in Zengjiazhai Village, Yuchong Town, Dafang County, Guizhou Province, China (27.4232°N, 105.9309°E; ca 1530 m; Fig. 5). ***Paratypes***. • Six specimens from the same locality as the holotype. GZNUSLS202501288–290, 37.6–71.9 mm SL, GZNUSLS202501292–294, 48.1–52.6 mm SL, the collection data are the same as that of the holotype specimen.

#### Diagnosis.

*Triplophysa dafangensis* sp. nov. can be distinguished from all other species in the genus *Triplophysa* by the following combination of characteristics: body naked, without skin pigmentation, lateral line complete; eye reduced, with diameter of HL 5.3–7.1%; long outer rostral barbel reaching to or beyond posterior margin of posterior nostrils; distal margin of dorsal fin truncate; tip of pectoral fin not reaching to pelvic-fin origin, tip of pelvic fin beyond anus; dorsal fin 8, anal fin 7, and caudal fin 16 branched fin rays; vertebrae 43 (Fig. 7).

#### Description.

D iii, 8; A iii, 7; P i, 9; V i, 6; C 16; vertebrae 4+39 (*n* = 1).

***Body*** elongated; anterior trunk nearly cylindrical, posterior trunk laterally compressed. Head widest at operculum. Dorsal profile of head gradually rising; dorsal body profile elevated. Ventral profile from snout to origin of anal fin nearly straight. Body depth greatest just before origin of dorsal fin, slightly decreasing toward caudal base, body depth 12.9–15.8% of SL. Body smooth and scaleless; lateral line complete.

***Head*** conical; cephalic lateral-line system well developed, 24.3–26.6% of SL. Head width slightly greater than head height; head widest at operculum. Highest point of head at posterior region. Snout pointed, slightly blunt at tip; snout length slightly shorter than postorbital head length, accounting for a percentage of head length. Eyes degenerated; eye diameter 5.3–7.1% of head length; interorbital width 33.4–38.4% of head length. Anterior and posterior nostrils connected; anterior nostril located on apex of barbel-like nasal flap; posterior nostril opening slightly larger.

***Mouth*** inferior, U-shaped. Lips thick, with small papillae on upper lip. Median notch present on lower lip; upper lip entire. Three pairs of barbels well developed: inner rostral barbel, outer rostral barbel, and maxillary barbel. Outer rostral barbels longest, inner rostral barbels shortest. Inner rostral barbels not reaching anterior nostrils, 19.3–26.5% of head length; outer rostral barbels extending to or beyond posterior margin of posterior nostrils, 38.6–52.2% of head length; maxillary barbels reaching or slightly beyond posterior margin of eyes, 30.8–38.2% of head length. Posterior chamber degenerated; with the bony capsule forming a dumbbell shape.

Dorsal fin truncate posteriorly, 18.3–21.4% of SL. First branched ray longest, extending beyond vertical through anus, approaching vertical through anal fin origin. Anal fin well developed, posterior margin concave, 16.1–17.2% of SL; fin rays not reaching caudal-fin base. Pectoral fins well developed, 18.1–21.7% of SL, horizontally extended tips not reaching pelvic fin origin. Pelvic fins well developed, 14.7–16.6% of SL, horizontally extended origin slightly posterior to dorsal fin origin or opposite; tips extending beyond anus but not reaching anal fin origin; distance between anus and anal fin origin ~ 1 mm. Caudal fin deeply forked; upper lobe longer than lower lobe; tips slightly pointed. A low ridge-like adipose fin fold present on upper side of caudal peduncle.

#### Coloration.

In life, species pinkish-white in natural habitat, semi-transparent skin, body, and fin rays unpigmented, no markings (Fig. 8). After fixation in formalin, specimen pale yellow, body, and fin rays remain unpigmented (Fig. 6).

#### Sexual dimorphism.

No secondary sexual characteristics were observed in the collected specimens.

#### Distribution.

This new species was harvested from an underground river in a karst cave in Yuchong Township, Dafang County, which is halfway up a mountain near the Youshan River Scenic Area (Fig. 5). Local inhabitants have accessed the cave. The underground river is connected to the Erdao River, a tributary of the Chishui River. This is the first occurrence of a stygobitic species of *Triplophysa* within the Chishuihe River basin, indicating a healthy ecological environment in the region.

#### Etymology.

The specific name refers to the type locality (county) of the new species: Dafang County and the Latin suffix (ensis). We propose the Chinese name ‘Dà fāng Gāo yuán qiū’ (大方高原鳅).

##### Molecular phylogenetic analysis

**Figure 9.**
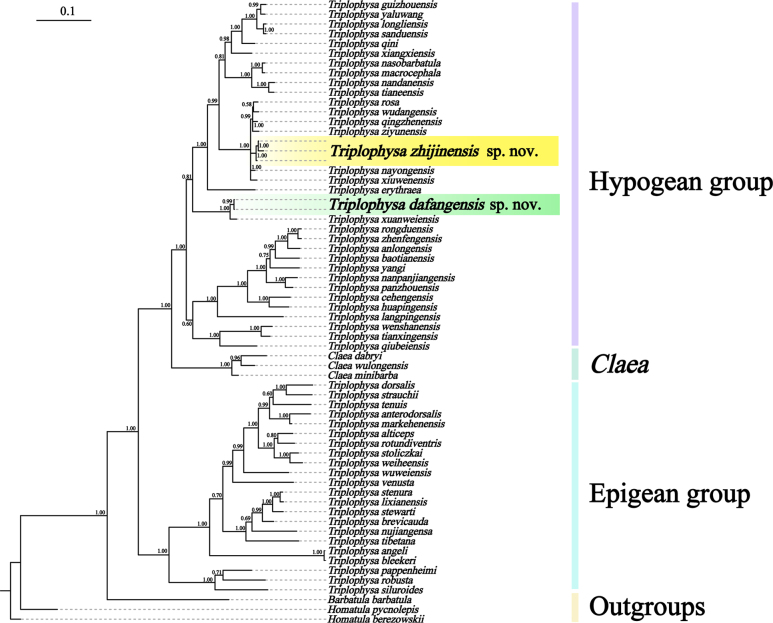
Phylogeny of some species of *Triplophysa*, *Claea* and three outgroup species based on Bayesian inference (BI) methods using mitochondrial Cyt *b* gene sequences. The BI posterior probabilities are shown at the nodes.

A phylogenetic analysis was performed based on Cyt *b* gene sequences from 61 species, including the two newly described species in this study. The BI tree revealed generally high posterior support across major nodes (Fig. 9). Both *Triplophysa zhijinensis* sp. nov. and *Triplophysa dafangensis* sp. nov. were placed within the cave-dwelling clade of *Triplophysa*, indicating their affinity with stygobitic lineages. However, the two species were not recovered as sister taxa and instead occupied distinct and well-supported lineages within the clade. *Triplophysa zhijinensis* sp. nov. formed a well-supported group with *T. nayongensis*, *T. xiuwenensis*, *T. ziyunensis*, *T. qingzhenensis*, *T. wudangensis*, and *T. rosa*, with a Bayesian posterior probability (BPP) of 1.00. In contrast, *Triplophysa dafangensis* sp. nov. was recovered as the sister species to *T. xuanweiensis*, with a BPP of 1.00. Genetic distances calculated under the Kimura 2-Parameter (K2P) model showed that *Triplophysa zhijinensis* sp. nov. differed from other cave-dwelling *Triplophysa* species by 2.3%–13.5%, while *Triplophysa dafangensis* sp. nov. differed from them by 1.6%–14.0%. The distance between the two new species was 13.3% (Table 4).

**Table 4.** Kimura-2-Parameter genetic distance (in %) among 20 cave species of *Triplophysa* based on Cyt *b* gene sequences.

		1	2	3	4	5	6	7	8	9	10	11	12	13	14	15	16	17	18	19
1	*T. dafangensis* sp. nov.																			
2	*T. zhijinensis* sp. nov.	13.3																		
3	*T. erythraea*	12.5	12.5																	
4	*T. guizhouensis*	12.8	10.0	11.8																
5	*T. longliensis*	12.9	10.2	12.1	2.8															
6	*T. macrocephala*	14.0	11.4	13.3	10.5	10.4														
7	*T. nasobarbatula*	13.0	11.1	13.4	10.4	10.2	0.7													
8	*T. nandanensis*	13.5	11.5	13.9	11.0	11.5	5.1	5.2												
9	*T. nayongensis*	12.1	2.3	12.7	9.8	10.4	10.5	10.2	10.8											
10	*T. qingzhenensis*	13.1	2.6	12.7	10.0	10.3	11.0	10.6	11.3	1.8										
11	*T. qini*	12.8	10.4	11.9	5.5	5.7	10.1	10.2	11.5	10.0	9.9									
12	*T. rosa*	13.0	2.7	13.1	10.2	10.6	11.3	11.0	11.8	1.8	1.5	10.3								
13	*T. sanduensis*	12.9	10.4	12.4	2.9	0.5	10.2	10.1	11.6	10.5	10.3	5.8	10.6							
14	*T. tianeensis*	13.7	11.8	13.2	11.4	12.0	5.4	5.5	1.8	11.2	11.2	11.2	12.0	12.1						
15	*T. wudangensis*	12.4	2.8	12.7	10.3	10.5	11.4	11.0	11.7	1.8	1.7	10.5	1.5	10.6	11.7					
16	*T. xiangxiensis*	12.8	10.2	13.0	9.0	8.4	9.1	9.0	10.7	9.4	9.7	6.4	9.8	8.7	10.0	10.1				
17	*T. xiuwenensis*	13.3	2.5	12.7	10.4	10.4	11.7	11.3	11.4	2.0	2.2	10.3	2.4	10.9	12.0	2.4	9.1			
18	*T. xuanweiensis*	1.6	13.5	13.5	13.1	13.3	12.8	12.7	13.1	12.2	13.0	12.8	13.1	13.3	13.4	12.5	12.8	13.4		
19	*T. yaluwang*	12.3	9.7	11.6	1.4	2.5	10.2	10.1	10.6	9.8	9.7	5.6	10.1	2.6	11.1	9.9	9.0	10.2	12.6	
20	*T. ziyunensis*	12.0	3.0	12.4	10.6	10.9	11.1	10.7	11.6	1.6	1.7	10.5	1.5	10.9	11.5	1.4	10.1	2.4	12.1	10.5

## Discussion

The detailed distinguishing characteristics between the two new species and 46 cave-dwelling species of this genus are shown in Table 3. *Triplophysa zhijinensis* sp. nov. can be distinguished from its congeners by skin pigmentation presence in dorsal (vs skin pigmentation absence in *T. aluensis*, *T. baishuijiangensis*, *T. cehengensis*, *T. erythraea*, *T. fengshanensis*, *T. gejiuensis*, *T. langpingensis*, *T. posterodorsalus*, *T. qini*, *T. qiubeiensis*, *T. shilinensis*, *T. tianlinensis*, *T. xiangxiensis*, *T. xuanweiensis*, *T. yangi*); *Triplophysa zhijinensis* sp. nov can be distinguished from *T. anlongensis*, *T. baotianensis*, *T. flavicorpus*, *T. guizhouensis*, *T. huapingensis*, *T. longipectoralis*, *T. longliensis*, *T. luochengensis*, *T. macrocephala*, *T. nandanensis*, *T. nanpanjiangensis*, *T. nasobarbatula*, *T. panzhouensis*, *T. rongduensis*, *T. sanduensis*, *T. tianeensis*, *T. tianxingensis*, *T. wenshanensis*, *T. xiangshuingensis*, *T. xingyiensis*, *T. yaluwang*, *T. yunnanensis*, *T. zhenfengensis*, by eye reduced, diameter of HL with 1.3/2.9%. *Triplophysa zhijinensis* sp. nov. can be distinguished from *T. anshuiensis* by CPL/CPD (1.59 vs 2.0–2.9); from *T. xichouensis* by anal-fin rays (iii, 5–6 vs ii, 6).

*Triplophysa zhijinensis* sp. nov. can be distinguished from *T. xiuwenensis* by skin pigmentation presence (vs skin pigmentation absence), eye diameter of HL (absent or 1.3/2.9% vs 4.6–6.7%), and vertebrae (4+35 in *Triplophysa zhijinensis* sp. nov. vs 4+37 in *T. xiuwenensis*). *Triplophysa zhijinensis* sp. nov. can be distinguished from *T. ziyunensis* by eye diameter of HL (absent or 1.3/2.9% vs 2.4–4.9%), interorbital width of HL (35.1/35.3% vs 22.3–26.2%), pectoral-fin rays (i, 9 vs i, 10) and caudal fin rays (14–15 vs 16). *Triplophysa zhijinensis* sp. nov. can be distinguished from *T. qingzhenensis* by interorbital width of HL (35.1/35.3% vs 25.1–30.4%), vertebrae (4+35 in *Triplophysa zhijinensis* sp. nov. vs 4+36 in *T. qingzhenensis*) and pelvic-fin rays (ii, 5–6 vs i, 5). *Triplophysa zhijinensis* sp. nov. can be distinguished from *T. wudangensis* by eye diameter of HL (absent or 1.3/2.9% vs 5.1–6.5%), vertebrae (4+35 in *Triplophysa zhijinensis* sp. nov. vs 4+34 in *T. wudangensis*), and 9 branched pectoral-fin rays (vs 8). *Triplophysa zhijinensis* sp. nov. can be distinguished from *T. rosa* by skin pigmentation presence (vs skin pigmentation absence), eye diameter of HL (absent or 1.3/2.9% vs eye absent), 9 branched pectoral-fin rays (vs 12) and dorsal-fin rays (iii, 8 vs iii, 9). *Triplophysa zhijinensis* sp. nov. can be distinguished from *T. nayongensis* by skin pigmentation presence (vs skin pigmentation absence), eye diameter of HL (absent or 1.3/2.9% vs eye absent), tip of pelvic fin not reaching anus (vs tip of pelvic fin reaching anus).

*Triplophysa dafangensis* sp. nov. can be distinguished from its congeners by skin pigmentation absence (vs skin pigmentation presence in *T. anlongensis*, *T. anshuiensis*, *T. baotianensis*, *T. flavicorpus*, *T. guizhouensis*, *T. huapingensis*, *T. longipectoralis*, *T. longliensis*, *T. luochengensis*, *T. macrocephala*, *T. nandanensis*, *T. nanpanjiangensis*, *T. nasobarbatula*, *T. panzhouensis*, *T. qingzhenensis*, *T. rongduensis*, *T. sanduensis*, *T. tianeensis*, *T. tianxingensis*, *T. wenshanensis*, *T. wudangensis*, *T. xiangshuingensis*, *T. xichouensis*, *T. xingyiensis*, *T. yaluwang*, *T. yunnanensis*, *T. zhenfengensis*, *T. ziyunensis*); *Triplophysa dafangensis* sp. nov. can be further distinguished from *T. anlongensis*, *T. baotianensis*, *T. guizhouensis*, *T. huapingensis*, *T. luochengensis*, *T. nandanensis*, *T. nanpanjiangensis*, *T. panzhouensis*, *T. qingzhenensis*, *T. rongduensis*, *T. sanduensis*, *T. tianeensis*, *T. tianxingensis*, *T. wenshanensis*, *T. wudangensis*, *T. xiangshuingensis*, *T. xingyiensis*, *T. yunnanensis*, *T. zhenfengensis* by tip of pelvic fin reaching anus (vs tip of pelvic fin not reaching anus); *Triplophysa dafangensis* sp. nov. can be further distinguished from *T. flavicorpus* by CPL/CPD (2.3–2.8 vs 1.7); from *T. longipectoralis*, *T. longliensis*, *T. nasobarbatula* by eye diameter of HL (5.3–7.1%); from *T. macrocephala* by CPL/CPD (2.3–2.8 vs 1.95); from *T. xichouensis* by eye diameter of HL (5.3–7.1% vs absent); from *T. yaluwang* and *T. ziyunensis* by vertebrae (4 + 39). *Triplophysa dafangensis* sp. nov. can be distinguished from *T. erythraea*, *T. fengshanensis*, *T. gejiuensis*, *T. posterodorsalus*, *T. qini*, *T. qiubeiensis*, *T. rosa*, *T. shilinensis*, *T. tianlinensis*, *T. xiangxiensis* by eye diameter of HL (5.3–7.1% vs absent). *Triplophysa dafangensis* sp. nov. can be distinguished from *T. aluensis* by interorbital width of HL (33.4–38.4% vs 22.2%); from *T. baishuijiangensis* by vertebrae (4 + 39 vs 4 + 34) and degenerated posterior chamber of air bladder (vs developed); from *T. cehengensis* by eye diameter of HL (5.3–7.1% vs 1.5–2.2%); from *T. langpingensis* by CPL/CPD (2.3–2.8 vs 1.55); from *T. xiuwenensis* by tip of pelvic fin reaching anus (vs tip of pelvic fin not reaching anus); from *T. yangi* by CPL/CPD (2.3–2.8 vs 1.7–1.9); from *T. nayongensis* by eye diameter of HL (5.3–7.1% vs absent) and tip of pelvic fin reaching anus (vs tip of pelvic fin not reaching anus).

*Triplophysa dafangensis* sp. nov. can be distinguished from *Triplophysa zhijinensis* sp. nov. by skin pigmentation absence (vs skin pigmentation presence), eye diameter of HL (5.3–7.1% vs absent or 1.3/2.9%), vertebrae (4+39 in *Triplophysa dafangensis* sp. nov. vs *Triplophysa dafangensis* sp. nov.) and pelvic-fin rays (i, 6 vs ii, 5–6). *Triplophysa dafangensis* sp. nov. can be distinguished from *T. xuanweiensis* by eye diameter of HL (5.3–7.1% vs eye absent), posterior chamber of air bladder (degenerated vs well developed), pectoral-fin rays (i, 9 vs i, 10–12), 14–16 branched caudal-fin rays (vs 17–18) and CPL/CPD (2.3–2.8 vs 2.0).

Like other cave-dwelling *Triplophysa*, the new species shares morphological traits commonly associated with cave adaptation. These features are widely considered evolutionary adaptations to permanent darkness and resource-limited subterranean habitats (Zhao et al. 2011). Additionally, both new species show notable intraspecific variation in pigmentation and eye development, ranging from moderately pigmented individuals with eyespots to fully de-pigmented and eyeless forms. This phenotypic diversity indicates that the genetic mechanisms underlying pigmentation and eye development have not been completely lost, there is clear evidence for selective pressures operating on genes associated with cave-derived regressive traits, such as eye development and pigmentation (Moran et al. 2023). Notably, once male secondary sexual characteristics appear, they remain stable and irreversible, indicating strong selective pressures linked to reproduction in cave environments (Zhu 1989). These findings contribute to the growing knowledge of subterranean ichthyofauna and offer valuable insights into the evolutionary and biogeographical processes shaping biodiversity in karst ecosystems.

Molecular phylogenetic analyses consistently reveal that *Triplophysa dafangensis* sp. nov. and *Triplophysa zhijinensis* sp. nov. represent two distinct evolutionary lineages, further validating the validity of the two new species. Additionally, the genetic distance calculated using the molecular marker Cyt *b* also distinguishes these species from other members of the *Triplophysa* hypogean group. Phylogenetic analyses show that the two new species belongs to the hypogean group of *Triplophysa* and that this hypogean group is monophyletic. Whereas cave-dwelling *Triplophysa* species form a sister group with *Claea*, together clustering with the epigean group of *Triplophysa* (Zhang et al. 2024). This indicates that *Triplophysa* is not monophyletic as currently defined, underscoring the need for further systematic revision of the genus based on broader sampling and genomic evidence (Šlechtová et al. 2025).

The discovery of *Triplophysa dafangensis* sp. nov. in the Chishuihe River is biogeographically significant, representing the first record of *Triplophysa* cavefish in this river system (Liu et al. 2020). In contrast, *Triplophysa zhijinensis* sp. nov. is distributed in the Wujiang River, a tributary of the Yangtze River, where cave fish have been previously reported, indicating that the Wujiang River might be a significant distribution area for *Triplophysa* cave-dwelling species (Chen and Yang 2005; Liu et al. 2022). The contrasting geomorphological and hydrological settings of the Chishuihe (Danxia–karst composite, with less developed subterranean systems) and the Wujiang (typical karst plateau with extensive caves and underground rivers) further highlight the complexity of dispersal pathways in this region (Chen et al. 2023).

## Supplementary Material

XML Treatment for Triplophysa zhijinensis

XML Treatment for Triplophysa dafangensis

## Appendix 1

Comparative data for the following species were acquired by specimen examination:

***Triplophysa anlongensis*** (*n* = 3): China: Guizhou: Anlong County: Xinglong Town: GZNUSLS202501337–GZNUSLS202501339.

***Triplophysa baotianensis*** (*n* = 3): China: Guizhou: Panzhou City: Baotian Town: GZNUSLS202201086–GZNUSLS202201088.

***Triplophysa cehengensis*** (*n* = 3): China: Guizhou: Ceheng County: Rongdu Town: GZNUSLS202407006–GZNUSLS202407008.

***Triplophysa erythraea*** (*n* = 7): China: Hunan: Fenghuang County: Heku Town: GZNUSLS202503019–GZNUSLS202503025.

***Triplophysa guizhouensis*** (*n* = 10): China: Guizhou: Huishui County: Baijin Town: GZNUSLS202502020–GZNUSLS202502029.

***Triplophysa huapingensis*** (*n* = 13): China: Guangxi: Baise City: Leye County: Chaoyang Town: GZNUSLS210702498–GZNUSLS210702510.

***Triplophysa langpingensis*** (*n* = 1): China: Guangxi: Baise City: Tianlin County: Langping Town: GZNUSLS202108205.

***Triplophysa longliensis*** (*n* = 3): China: Guizhou: Longli County: Baisheng Village: GZNUSLS022072266–GZNUSLS022072268.

***Triplophysa nasobarbatula*** (*n* = 11): China: Guizhou: Libo County: Maolan Town: GZNUSLS202001339–GZNUSLS202001349.

***Triplophysa nanpanjiangensis*** (*n* = 17): China: Yunnan: Qujing City: Zhanyi District: GZNUSLS202407034–GZNUSLS202407050.

***Triplophysa panzhouensis*** (*n* = 4): China: Guizhou: Panzhou City: Hongguo Town: GZNUSLS202304280–GZNUSLS202304283.

***Triplophysa qingzhenensis*** (*n* = 15): China: Guizhou: Guiyang City: Qingzhen City: GZNUSLS202110074–GZNUSLS202110088.

***Triplophysa qiubeiensis*** (*n* = 5): China: Yunan: Qiubei County: Shuanglongying Town: GZNUSLS202109028–GZNUSLS202109032.

***Triplophysa rongduensis*** (*n* = 5): China: Guizhou: Ceheng County: Rongdu Town: GZNUSLS202407001–GZNUSLS202407005.

***Triplophysa rosa*** (*n* = 5): China: Chongqing City: Wulong District: Jiangkou Town: GZNUSLS202105001–GZNUSLS202105005.

***Triplophysa sanduensis*** (*n* = 4): China: Guizhou: Sandu County: Zhonghe Town: GZNUSLS202201082–GZNUSLS202201085.

***Triplophysa tianeensis*** (*n* = 11): China: Guangxi: Tian’e County: Bala Township: GZNUSLS202504009–GZNUSLS202504019.

***Triplophysa tianxingensis*** (*n* = 1): China: Yunan: Guangnan County: Zhulin Town: GZNUSLS202307046.

***Triplophysa wudangensis*** (*n* = 3): China: Guizhou: Guiyang City: Wudang District: GZNUSLS202304284, GZNUSLS202410001–GZNUSLS202410002.

***Triplophysa xiangshuingensis*** (*n* = 2): China: Yunan: Kunming City: Shilin County: Lufu Town: GZNUSLS202304270–GZNUSLS202304271.

***Triplophysa xingyiensis*** (*n* = 9): China: Guizhou: Xingyi City: Zhengtun Town: GZNUSLS202507016, GZNUSLS202407018, GZNUSLS202407024, GZNUSLS202507023–GZNUSLS202507027.

***Triplophysa xiuwenensis*** (*n* = 4): China: Guizhou: Xiuwen County: Dashi Buyi Township: GZNUSLS202306086, GZNUSLS202306088–090.

***Triplophysa yangi*** (*n* = 3): China: Yunnan: Shizong County: Wulong Township: GZNUSLS202501302–GZNUSLS202501304.

***Triplophysa yunnanensis*** (*n* = 6): China: Yunnan: Yiliang County: Jiuxiang Yi and Hui Ethnic Township: GZNUSLS202304260–GZNUSLS202304265.

***Triplophysa zhenfengensis*** (*n* = 5): China: Guizhou: Zhengfeng County: Longchang Town: GZNUSLS202311039–GZNUSLS202311043.

The data for the following species were obtained from literature: *T. aluensis* (Li and Zhu 2000), *T. anshuiensis* (Wu et al. 2018), *T. baishuijiangensis* (Shi et al. 2025), *T. fengshanensis* (Lan et al. 2013), *T. flavicorpus* (Yang et al. 2004), *T. gejiuensis* (Chu and Chen 1979), *T. longipectoralis* (Zheng et al. 2009), *T. luochengensis* (Li et al. 2017a), *T. macrocephala* (Yang et al. 2012), *T. nandanensis* (Lan et al. 1995), *T. nayongensis* (Dong et al. 2026), *T. posterodorsalus* (Ran et al. 2006), *T. qini* (Deng et al. 2022), *T. shilinensis* (Chen et al. 1992), *T. tianlinensis* (Li et al. 2017b), *T. wenshanensis* (Cao et al. 2025), *T. xiangxiensis* (Yang et al. 1986), *T. xichouensis* (Liu et al. 2017), *T. xuanweiensis* (Lu et al. 2022), *T. yaluwang* (Lan et al. 2024), *T. ziyunensis* (Lan et al. 2024).

## Additional information

### Artificial Intelligence (AI) use

The authors accept full responsibility for the content of the manuscript, including the disclosure of any use of AI.

No AI tools were used in the preparation of this manuscript.

### Author ORCIDs

T.-T. Zhu https://orcid.org/0000-0003-4178-2785

F.-H. Yuan https://orcid.org/0009-0004-5112-0683

R.-Y. Zhang https://orcid.org/0000-0002-3647-1416

Y.-H. Zhao https://orcid.org/0000-0002-4615-596X

### Data availability

All of the data that support the findings of this study are available in the main text.
